# Mechanistic Investigation on ROS Resistance of Phosphorothioated DNA

**DOI:** 10.1038/srep42823

**Published:** 2017-02-20

**Authors:** Tingting Wu, Qiang Huang, Xiao-Lei Wang, Ting Shi, Linquan Bai, Jingdan Liang, Zhijun Wang, Zixin Deng, Yi-Lei Zhao

**Affiliations:** 1 State Key Laboratory of Microbial Metabolism, Joint International Research Laboratory of Metabolic & Developmental Sciences, MOE-LSB and MOE-LSC, School of Life Sciences and Biotechnology, Shanghai Jiao Tong University, Shanghai, 200240, China

## Abstract

Phosphorothioated DNA (PT-DNA) exhibits a mild anti-oxidant property both *in vivo* and *in vitro*. It was found that 8-OHdG and ROS levels were significantly lower in *dnd*+ (i.e. *S*^+^) *E. coli.*, compared to a *dnd*− (i.e. *S*^−^) strain. Furthermore, different from traditional antioxidants, phosphorothioate compound presents an unexpectedly high capacity to quench hydroxyl radical. Oxidative product analysis by liquid chromatography-mass spectrometry and quantum mechanistic computation supported its unique anti-oxidant characteristic of the hydroxyl selectivity: phosphorothioate donates an electron to either hydroxyl radical or guanine radical derived from hydroxyl radical, leading to a PS^•^ radical; a complex of PS^•^ radical and OH^−^ (i.e. the reductive product of hydroxyl radical) releases a highly reductive HS^•^ radical, which scavenges more equivalents of oxidants in the way to high-covalent sulphur compounds such as sulphur, sulphite and sulphate. The PS-PO conversion (PS and PO denote phosphorus-sulphur and phosphorus-oxygen compounds, respectively) made a switch of extremely oxidative OH^•^ to highly reductive HS^•^ species, endowing PT-DNA with the observed high capacity in hydroxyl-radical neutralization. This plausible mechanism provides partial rationale as to why bacteria develop the resource-demanding PT modification on guanine-neighboring phosphates in genome.

DNA Phosphorothioation is a novel type of chemical modification originally discovered in *Streptomyces* species and then determined genetically in many kinds of bacteria^1^,^2^,^3^. The chemical modification in gene of *Streptomyces lividans* is found to be *Rp* stereo-specific and sequence-selective, which was partially rationalized theoretically^4^,^5^,^6^ and verified experimentally^7^,^8^; moreover, a mobile five-membered gene cluster *dnd*ABCDE^9^,^10^,^11^ commits the chemical modification on bacterial DNA. Basically, two biological functions were proposed for the physiological DNA modification^12^,^13^,^14^,^15^,^16^. Molecular recognition between PT-DNA motif and specific proteins strongly endorsed its restriction response in host bacteria^12^,^13^,^14^; for example, certain species (not all *dnd*+ bacteria) such as *S. enterica* serovar Cerro 87, possesses a downstream gene cluster *dndFGHI* after *dndA-E* to encode a restriction-enzyme system that distinguishes whether phosphorothioation occurred at the specific site in DNA. Besides, anti-oxidantivity of phosphorothioated DNA (PT-DNA) was aware in the previous experiment^15^, but then in the deep dispute: the original discovery of anti-oxidant benefit might be flawed by the use of wild-type bacteria possessing both PT modification genes and PT-dependent restriction genes; knocking out the PT modification genes would lead to higher sensitivity toward oxidative stress – it might be false positive from side-affect of remaining restriction enzymes in the wild-type bacteria^14^. Recently, a following-up paper has been published in *Frontiers in Microbiology* by Dai *et al*., confirming that the phantomlike resistance in wide type *Streptomyces lividans* to oxidative stress is relevant to PT-DNA directly, neither other *dnd* proteins nor RNA-related regulation^16^. However, more questions need addressing for the elusive biological effect, in particular at the level of biochemistry and molecular biology - For example, how are the rare copies of PT modifications able to conveys special *in vivo* resistance to oxidative stress? – it should be noted that the chemical modification occurs at guanine-neighbouring sites such as GpsA, GpsT, and CpsC (complementary of GG) at a relatively low frequency of 300~3000/10^6^ nt^1^,^2^. How to explain the fact that the *in vitro* PT oxidation led to both sulphur removal and DNA backbone cleavage? Thus, the earlier works about the PT antioxidant activity were still preliminary and conflicting, mainly for lack of detailed mechanistic investigation in rationale on the promising hypothesis that the sparse PT sites meet anti-oxidant needs of a long nucleobase sequence.

Indeed, DNA oxidative damage is usually caused by highly reactive oxygen species (ROS), such as singlet oxygen, superoxide, hydrogen peroxide, and hydroxyl radical^17^. Many chemical modifications in DNA damage (*e.g.* DNA cleavage, DNA-protein cross links, purine oxidation, etc.) stem from the reactions with ROS, in particular with hydroxyl radical (HO^•^). Hydroxyl radical is very rare in a living cell, but extremely reactive, scrambling electrons and hydrogen atoms from protein and metabolites in a diffusive manner, and even from stable biomolecules such as DNA and lipids. Both the heterocyclic base and sugar-phosphate backbone are vulnerable, in particular via electron abstraction on guanine base and hydrogen abstraction on ribose 5′-C. If the nucleobase-repair systems are not able to immediately regenerate intact nucleobase, a mutation in genome would result from erroneous base pairing during replication or, consequently, DNA cleavage would occur consequently. It is also believed that metal ions such as iron and copper would enhance hydroxyl radical generation via Fenton chemistry^18^.

In short, DNA oxidative damage seems to begin with one-electron loss and formation of a radical cation “hole” at the encounter position^19^. The hole hops reversibly through electronic structure along the DNA helix, trapped at a guanine-rich place as a form of guanine radical cation (G^•+^)^20^. Hydrolysis of G^•+^ releases 8-hydroxy-2′-deoxyguanosine (8-OHdG), upon consequential DNA repairing *in vivo*. So 8-OHdG is an effective biomarker for DNA oxidative damage^21^.

Fischer *et al*. have also reported *in vivo* antioxidant capacity of 5′-PT-dNTP in drug development for neurodegenerative disorders, but as attributing to metal-ion coordination and Fenton inhibition^22^. In contrast, low molecular-weight free thiols such as glutathione (GSH), cysteine (CYS), and homocystine can ameliorate oxidative damage in an alternative fashion, via an oxidative dimerization towards RSSR^23^.

In this work we measured the both the 8-OHdG and ROS intracellular levels of *dnd*+ (*S*^+^) and *dnd*− (*S*^−^) strains of *E. coli* without intervention of restriction genes *dndFGHI* to verify the anti-oxidant property of PT-DNA. Then we revealed its anti-oxidant mechanism with *in vitro* oxidation of pJTU1238 plasmid and phosphorothioate dinucleotide, using gel electrophoresis, circular dichroism spectroscopy, high performance liquid chromatography - mass spectroscopy, and quantum chemistry. Here we propose a unique anti-oxidant mechanism, different from metal-ion coordination^18^ and sulfur-sulfur dimerization^23^ in the literatures.

## Results

### *In vivo* Anti-oxidant Effect

**Figure 1:**
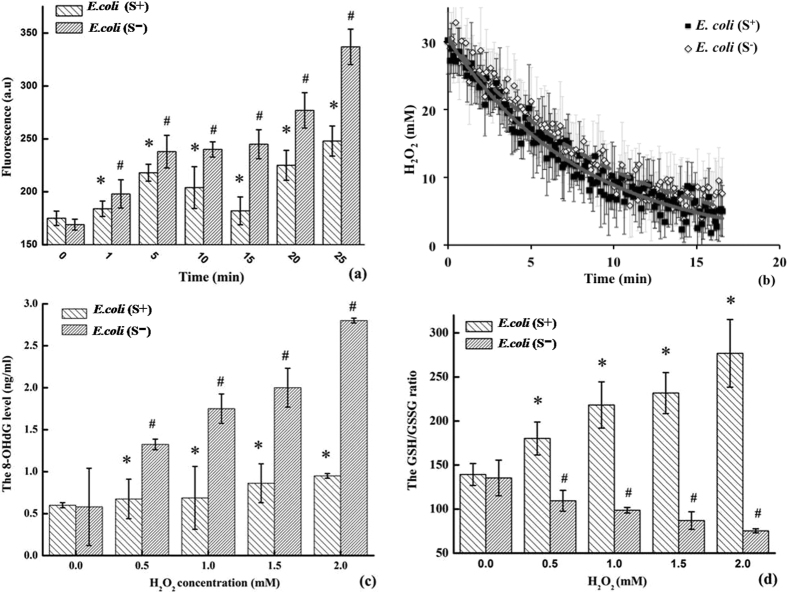
The *in vivo* antioxidant response. (**a**) The intracellular ROS levels measured by the DCF fluorescent intensity at excitation of 485 nm and emission of 528 nm, treated with 0.5 mM H_2_O_2_; (**b**) the time-resolved peroxide concentrations monitored with Raman vibration at 876 cm^−1^ and initial concentration of 30 mM H_2_O_2_; (**c**) the 8-OHdG level and (**d**) the GSH:GSSG ratio in the samples treated for 4 hours with moderate concentrations of H_2_O_2_ (the treatments included 6.0 μM FeSO_4_ as Fenton’s catalyst; asterisk and octothorp mark the statistical significance, p < 0.05).

Figure 1a shows DCF fluorescence of *dnd*+ (S^+^) and *dnd*− (S^−^) *E. coli* strains over time under oxidative stress mimicked with Fenton’s reagent of 6 μM FeSO_4_ and 0.5 mM H_2_O_2_. Compared to the starting point, the intracellular ROS level represented by DCF fluorescence climbed up by 18.0 ± 8.4, 40.9 ± 10.0, 42.7 ± 4.7, 45.2 ± 7.8, 62.1 ± 11.7, and 94.8 ± 10.2% in the *dnd*− culture, at 1, 5, 10, 15, 20, and 25 min, respectively. By contrast, the intracellular ROS accumulation in *E. coli* (S^+^) strain fluctuates at 3.8 ± 5.5, 22.5 ± 5.7, 14.9 ± 12.9, 27.3 ± 6.6, 40.5 ± 4.9, and 35.0 ± 12.7%, respectively.

Figure 1b shows the real concentration of hydrogen peroxide of *dnd*+ (S^+^) and *dnd*− (S^−^) *E. coli* strains over time under oxidative stress, using 30 mM H_2_O_2_ (similar to the experimental condition in ref. 16), monitored by the O-O Raman vibrational band at 876 cm^−1^. Accordingly, hydrogen peroxide consumed rapidly in an exponential-decay fashion for a variety of reductants in the living organisms, and the regression analysis of five replicas resulted in decay time constants of 8.5 ± 1.7 and 9.8 ± 1.9 min in the cases of the *S*^+^ and *S*^−^ cultures, respectively. This observation was in agreement with the previous experiments of wide type *S. lividans* and the *dnd*− mutant treated with 20 mM H_2_O_2_, reported by Dai *et al*. (as shown in Fig. S2 of ref. 16).

As shown in Fig. 1c, when the strains were charged with a moderate Fenton’s reagent, the 8-OHdG level arose about 12.5 ± 14.7~58.3 ± 2.3% in the *S*^+^ strain with increasing H_2_O_2_ concentrations from 0.5 to 2.0 mM; conversely, the values in the S^−^ strains rocketed up by 32.5 ± 3.3~180.0 ± 4.1%. In Fig. 1d, the measured GSH:GSSG ratio dropped down dramatically from 135.4 ± 20.2 to 75.4 ± 2.2 in the *E. coli (S*^−^) strain. Conversely, the values increased in the *E. coli (S*^+^) strains from 139.3 ± 12.5 to 276.8 ± 38.4. In literatures, the normal GSH:GSSG level of about 200 decreased to 10~1 under oxidative stress^24^, and a 2.5 fold increase of the 8-OHdG level was reported in 10 T½ cells in the presence of 0.1 mM H_2_O_2_^25^.

### *In vitro* Anti-oxidant Tests of Plasmid PT-DNA

**Figure 2:**
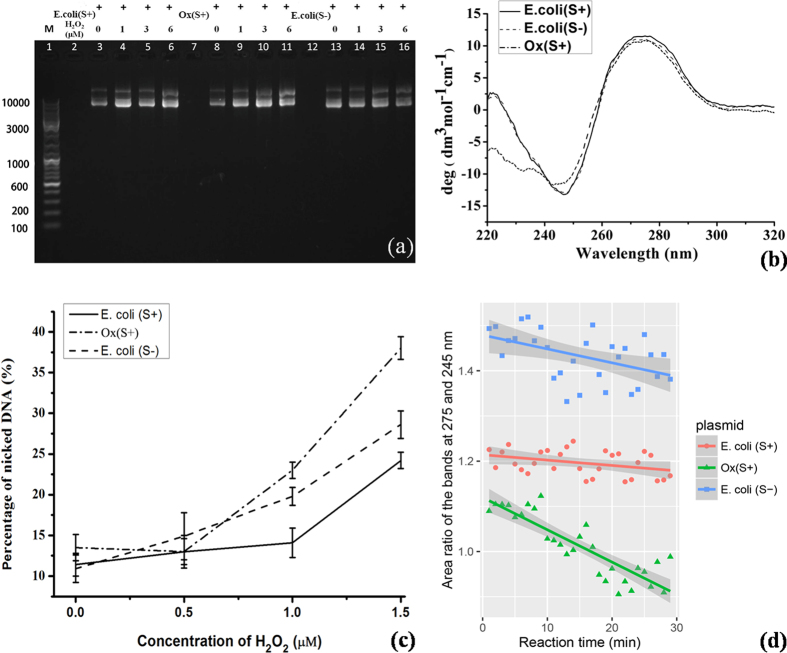
The *in vitro* antioxidant detections of the extracted plasmid DNA samples. (**a**) The image of plasmid DNA gel electrophoresis, where the 63 μg/mL DNA samples treated with H_2_O_2_ up to 1.5 μM for 12 hours; (**b**) the circular dichroism spectra of the DNA samples; (**c**) the quantitated nicked DNA percentage in total amount of DNA, upon the oxidation; (**d**) the quantitated helicity upon the oxidative damage at 1.0 μM H_2_O_2_.

Figure 2 showed the *in vitro* experimental results of plasmid oxidation with H_2_O_2_ concentrations in a range of 0.0 to 1.5 μM for 12 hours in the dark, with comparison to a ABTS pre-oxidized PT-DNA sample ([PT] ≈ 0.19 μM). It is almost indistinguishable in the image of gel electrophoresis (Fig. 2a) and the circular dichroism (CD) spectra (Fig. 2b) owing to the technical limitation; so digital quantitation on the two *in vitro* experiments was carried out.

Figure 2c show quantitation of the electrophoresis image by software image J^26^. Nicked circular DNA percentage in the total plasmid DNA (supercoil plus linear and nicked circular) was used to exhibit DNA oxidative damage^27^. The nicked-DNA percentage of PT plasmid slightly increased by 1.6 ± 1.6 and 2.6 ± 1.7% at 0.5 and 1.0 μM H_2_O_2_, respectively. However, the values were measured as 4.0 ± 2.9 and 8.9 ± 1.7% in the *dnd*− plasmid, and −0.4 ± 2.0 and 9.4 ± 1.6% in the pre-oxidized PT-DNA, respectively. All the three plasmid samples exhibited severe oxidative damage in the presence of 1.5 μM H_2_O_2_, with the nicked-DNA percentages of 12.7 ± 1.4, 17.7 ± 1.7, and 24.5 ± 1.6% for the PT-, normal plasmid, and pre-oxidized PT-, respectively.

Figure 2d shows quantitation of DNA conformational damage by oxidative stress, represented with a ratio of integrated areas under the positive band at 275 nm over the negative band at 245 nm in CD spectra. It has been reported that declining the positive ellipticity and inclining the negative ellipticity characterize oxidative damage on plasmid DNA^27^. For purpose of controlling errors from peak-wavelength shift, the integrated value in half-band width was computed and their ratios were used to denote DNA helicity. The ratios drop down by slopes of −1.2 ± 0.6, −3.1 ± 1.1, and −7.1 ± 0.8 (×10^−3^ min^−1^) for the PT-, normal, and pre-oxidized PT-DNA plasmid, respectively. On the other hand, the absolute values of ratio in the order of *dnd*− >  *dnd*+ >  pre-oxidized PT-DNA plasmid indicated that DNA oxidation damaged the secondary structure significantly while DNA phorosphorothioation disturbed the B-helical conformation slightly^5^.

### Comparison of PS, GSH, CYS, and vitC

Owing to the μM technical limitation of plasmid PT-DNA, diethyl phosphorothioate (**PS**) was used as a model compound of PT-DNA in the following *in vitro* comparative experiments with non-physiological oxidants ABTS•+ and DPPH• radicals. GSH, CYS, and vitC appeared to possess a similar scavenging capacity with reference to 46.7 μM ABTS•+ in 30 min, with the measured IC50 values of 5.1 ± 0.2, 5.9 ± 0.1, and 7.1 ± 0.1 μM, respectively. This value was 42.3 ± 1.2 μM for PS, six times higher than those of traditional antioxidants. Similarly, the IC50 values towards 200 μM DPPH in 30 min were determined as 36.5 ± 1.5, 31.2 ± 0.2, and 25.3 ± 0.4 μM for GSH, CYS, and vitC, respectively; while the IC50 value for PS was measured to be 230.7 ± 8.1 μM, also about 6 to 8 times higher than the others. Thus, in terms of IC50 value with reference to organic oxidants ABTS•+ and DPPH•, phosphorothioate exhibited a mild anti-oxidant activity, in agreement with its weak reductive potential.

**Figure 3:**
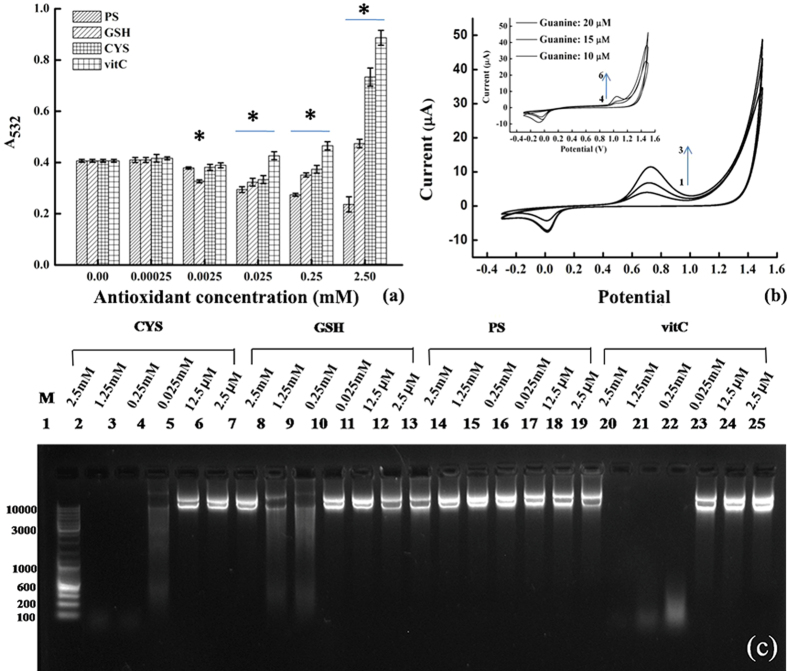
The antioxidant and pro-oxidant properties of phosphorothioate (PS), compared to the other antioxidants. (**a**) The ROS levels characterized by the amounts of TBARS with the absorbance at wavelength of 532 nm in 30 min, generated by Fenton’s reagent of 50 μM FeSO_4_ and 1.0 mM H_2_O_2_ at 37 °C and quenched with gradient antioxidant concentrations from 0.25 μM to 2.5 mM; (**b**) the cyclic voltammograms of the phosphorothioate model compound (lines **1**–**3**: 10, 15, and 20 μM, respectively) and 2′-deoxyguanosine (insert, lines **4**–**6**: 10, 15, and 20 μM, respectively), with potential voltages referred to a Ag/AgCl electrode; (**c**) the image of gel electrophoresis, in which 50 μg/mL plasmid DNA were pre-treated with the Fe(III)-modified Fenton’s reagent and gradient concentrations of antioxidants from 2.5 mM to 2.5 μM.

As shown in Fig. 3a, the ROS level characterized by the TBARS amount decreased by 0.0 ± 2.4, 7.3 ± 1.3, 29.3 ± 3.5, 34.2 ± 2.2, and 41.5 ± 12.5% at the PS concentrations of 0.25, 2.5, 25.0, 250.0, and 2500.0 μM, respectively. The values were 0.0 ± 4.9, 19.5 ± 1.8, 22.0 ± 6.3, and 14.6 ± 2.3% for GSH, and −2.4 ± 4.8, 7.3 ± 2.6, 19.5 ± 6.1, and 9.8 ± 5.4% for CYS, respectively. The corresponding ROS generation increased by 78.1 ± 5.5 and 14.6 ± 4.3% at 2500.0 μM in the case of GSH and CYS, respectively. Similar phenomena were observed with anti-oxidant vitC, in which the ROS generation increased by 4.9 ± 4.7, 14.6 ± 4.3, and 117.1 ± 3.4% at the concentrations of 25.0, 250.0, and 2500.0 μM, respectively.

According to electrochemical measurement (Fig. 3b), the reduction potential of PS (0.74 V vs Ag/AgCl) is lower than that of 2′-deoxyguanosine by about 0.28 V. The standard reduction potentials of guanine, adenine, thymidine, and cytosine were reported to be about 1.29, 1.42, 1.70, and 1.60 V^28^,^29^. So the standards reduction potential of phosphorothioate was estimated as 0.94 V after calibration, higher than that of Fe(III)/Fe(II) by 0.20 V, but lower than the G^•+^/G potential. Therefore, the pro-oxidant effect for these antioxidants was determined to conform to the order of CYS > vitC > GSH ≫ PS (Figs S3 and S4). Similarly, oxidative damage of plasmid DNA in the DNA lesion experiment can be used to test the pro-oxidant effect in the presence of Fe(III)-modified Fenton’s reagent^30^. PS gave no detectable pro-oxidativity up to 2.5 mM, while the other three antioxidants exhibited the DNA degradation at the relatively high concentrations (Figs 3c and S5).

### LCMS Characterization of Oxidative Products

**Figure 4:**
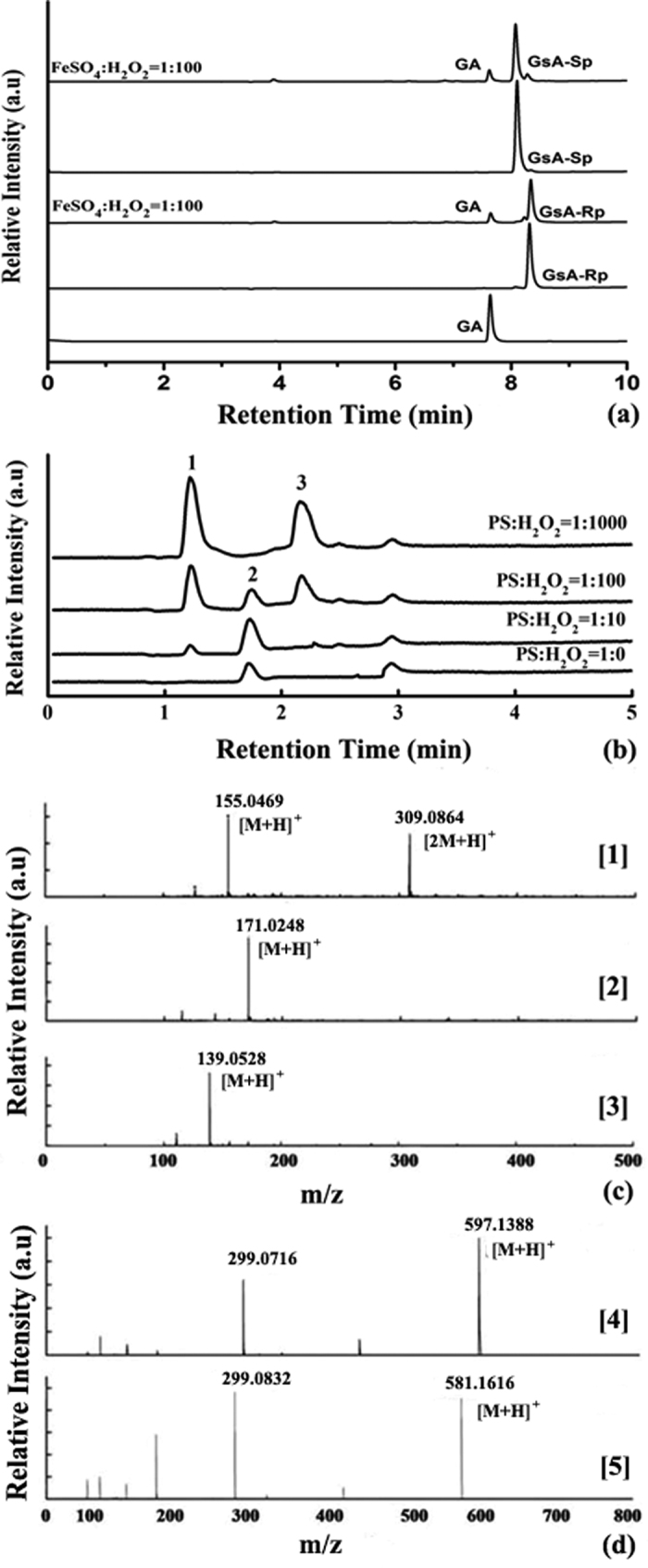
The HPLC and LCMS analysis of oxidative products of (**a**) the mixture of 20 μM GsA-Rp and GsA-Sp with 100 μM H_2_O_2_ plus 1 μM FeSO_4_, and (**b**) the mixture of 25 μM PS with the gradient H_2_O_2_ concentrations; (**c**) the high-resolution mass spectra of components 1 (**PO**), 2 (**PS**), and 3 (**PH**), respectively; (**d**) GsA and GA.

As shown in Fig. 4a, two stereochemical phosphorothioated dinucleotides GsA (retention time of *Rp-* configuration at 8.5 min and *Sp*- at 8.1 min) both led to GA dinucleotide (retention time at 7.5 min). As a comparison, Fig. 4b shows the H_2_O_2_-dependent PH formation (PH: hydrogen-phosphonate compound) in oxidation of diethyl phosphorothioate (PS) by gradient H_2_O_2_ concentration. The HPLC experiments showed two new peaks for the PS oxidation by H_2_O_2_ (Fig. 4b, peak 1 and 3). As shown in Fig. 4c, high-resolution mass spectrum analysis indicated that the substance of peak 1 was O,O-diethyl phosphate (**PO**, [M + H]^+^ m/z: *exp.* 155.0469, *ca.* 155.0473), peak 2 O,O-diethyl thiophosphate (**PS**, original reactant, [M + H]^+^ m/z: *exp.* 171.0248, *ca.* 171.0245), and peak 3 O,O-diethyl hydrogen-phosphonate (**PH**, [M + H]^+^ m/z: *exp.* 139.0528, *ca.* 139.0524), with retention times at 1.2, 1.7, and 2.2 min, respectively. The two oxidative products (**PO** and **PH**) were proportional to H_2_O_2_ concentration in the oxidation.

### *In silico* Justification

**Figure 5:**
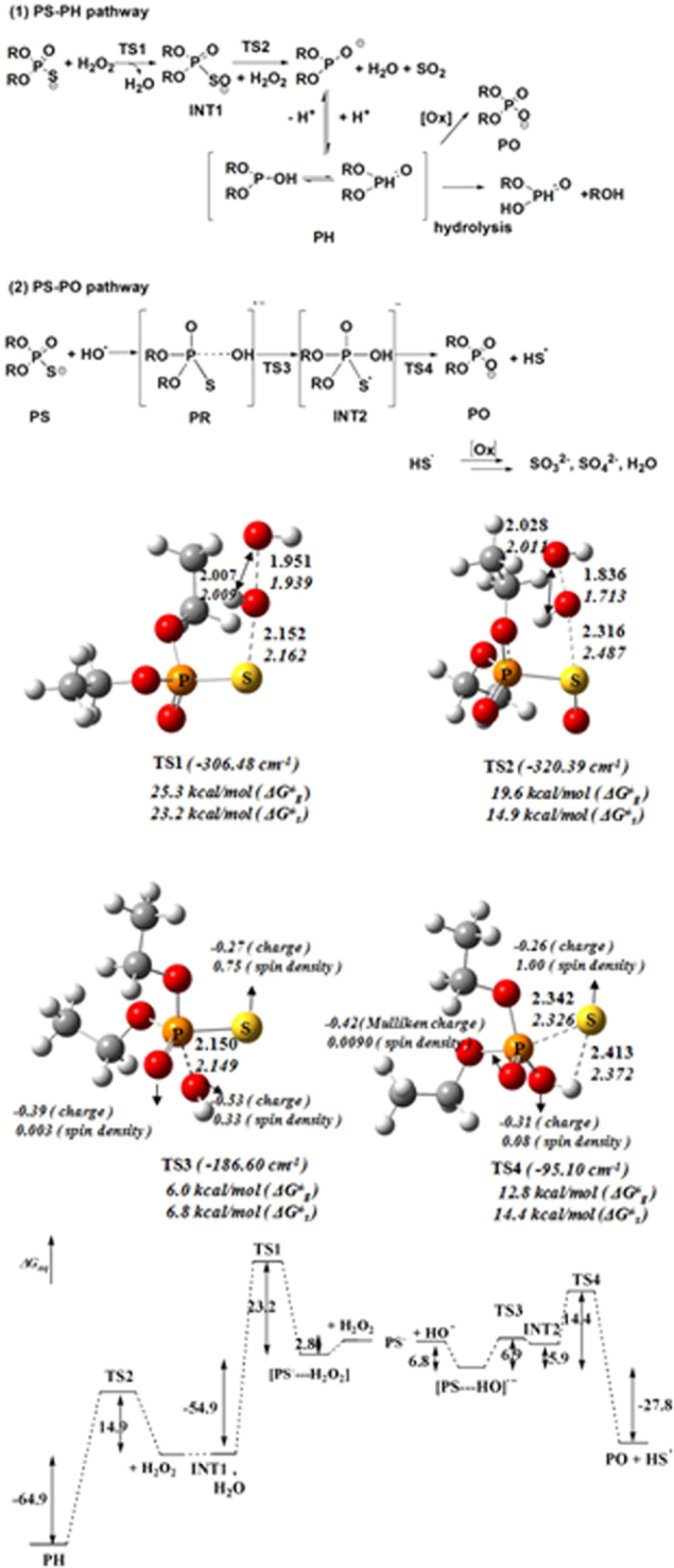
The PH and PO pathways, transition structures, and energy diagram of ROS-involving phosphorothioate oxidation at the M06 and B3LYP level of theory with the basis set of 6-311 + G(d, p). (Distances in angstrom, and energetics in kcal/mol).

We carried out intensive quantum chemical calculations for the reaction pathway from PS toward PH and PO (Table S1 and Fig. S8). Figure 5 shows the most plausible mechanism in which PH was generated from two oxygen-donations of H_2_O_2_. In the first step, one molecule of H_2_O_2_ reacts with PS to form PSO anion (INT1) and release one molecule of water, with a calculated barrier of 23.2 kcal/mol in aqueous solution. Then, another molecule of H_2_O_2_ reacts with PSO anion, releasing one molecule of sulphur dioxide (SO_2_) and one water molecule and then leading to PH generation. The reaction barrier for the second step was computed to be 14.9 kcal/mol.

**Figure 6:**
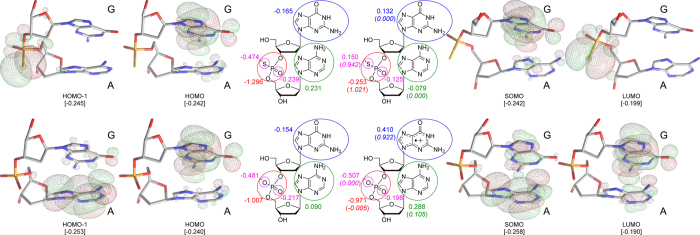
The frontier orbital surfaces, Mulliken charge, and spin density (parenthesized and italic, within the circled fragments) of GsA and GA molecules, in both the anionic and radical forms, calculated at the M06 level of theory.

By comparison to the PS-PH conversion, the most plausible mechanism for PS-PO conversion has a shortcut path in the presence of hydroxyl radical. Electron transfer from phosphorothioate anion to HO^•^ or G^•+^ seemed barrier-free for the extremely high reactivity of hydroxyl radical - a pre-reactive complex of [PS---OH]^−•^ was formed instantly. The radical anion complex transformed to a five-coordinate adduct (INT2) via phosphor hydroxylation, with a calculated barrier of 6.9 kcal/mol. Such an addition-elimination mechanism is similar to hydrolysis of organophosphorus pesticides^31^. The INT2 was higher in energy by 5.9 kcal/mol than the pre-reactive complex. INT2 underwent an elimination of hydrogen-sulphur radical (HS^•^), forming the normal phosphate with a calculated barrier of 14.4 kcal/mol. The reaction of PS plus HO^•^ towards PO plus HS^•^ was exothermic by 27.8 kcal/mol. Therefore, the overall reaction barrier for the PS-PO conversion was estimated to be 14.4 kcal/mol (ΔG^≠^) in an aqueous solution. As Fig. 6 shows, the frontier orbitals of GA and GsA were observed at the M06 (CPCM, solvent = water)/6-311 + G(d, p)//M06/6-31 G(d) level of theory.

## Discussion

The *in vivo* experiments indicated that the model organisms (*E. coli,* as shown in Fig. 1) of this study led to the consistent results as wild type *S. lividans (dnd*+, *i.e. S*^+^) and the knocking-out mutant (*dnd*−, *i.e. S*^−^) to oxidative stress reported by Dai *et al*.^16^. It should be acknowledged that the additional anti-oxidant capacity in the *S*^+^ strain isn’t major given that the decay time changed slightly for extracellular peroxide (12~13%) owing to coexistence of various anti-oxidant systems in the living organisms. Beside a slightly fast consumption of peroxide, it was first time to observe the intracellular ROS accumulation was suppressed by about 14~60% in the *S*^+^ strain, albeit with a sink at 10–15 min upon anti-ROS response time-delay as reported in the previous literature^32^. Dai *et al*. reported no significant up-regulation of anti-oxidant genes based on RNA-seq experiment of wide type *S. lividans* upon oxidative stress^16^, in addition to the consistent observation on *dndFGHI-*negative *E. coli* in this work, clarifying the previous suspicion whether the *dnd*− knocking-out would disturb *dndABCDE/FGHI* restriction-response system (“sick cell”). However, the intercellular ROS response exhibited dramatical difference between the *S*^+^ and *S*^−^ cultures: the intracellular levels of 8-OHdG biomarker for DNA oxidative damage dropped significantly and those of GSSG biomarker for overall oxidation lowered as well; on the other hand, the GSH levels were similar in the two strains - consistent with no thiol peroxidase up-regulation based on RNA-seq experiment under oxidative stress^16^. All these results testified an effective anti-oxidant bypass in the *S*^+^ strain to maintain a reductive atmosphere in cell, which may stem from PT-DNA itself.

pJTU1238 in the *E. coli* strains were used to solve the anti-oxidant biochemistry of PT-DNA. Plasmid DNA was extracted from the two *E. coli* strains and tested for anti-oxidant activity *in vitro* directly. A typical PT population was estimated as ~1/1000 nt in the *S*^+^ strain based on the previous deep sequencing^1^, so the PT concentration in a stock solution of extracted *dnd*+ plasmid with optical density of 4.0 at 260 nm is quantified as ~200 μg/mL in *ds*-DNA weight or ~0.6 μM in PT equivalent. Owing to no further endogenous PT regeneration after the *E. coli* digestion, the anti-oxidant capacity of plasmid PT was limited at the micromolar (μM) level, significantly lower than those *in vivo* oxidant levels in Fig. 1^15^,^33^. The plasmid experiment in Fig. 2d indicated that the oxidative damage occurred 2.6 times slower in the PT-DNA, and upon pre-oxidation, the pre-oxidized PT-DNA appeared loss of the additional anti-oxidant property in the *in vitro* anti-oxidant experiments, consistent with the results from the gel-image quantitation (Fig. 2c).

Although the anti-oxidant property of the extracted plasmid touched limit of determination at 1 μM H_2_O_2_, it is worthwhile to note that the low-frequency PT in genome is in agreement with economical efficiency in biology, due to the low-frequency of DNA oxidative damage in reality, e.g. usually ~1 lesion per 10000 nt upon *gamma* radiolysis^34^.

Intriguingly, the antioxidant effect of phosphorothioate compound showed unexpected selectivity to hydroxyl radical in the anti-oxidant chemistry, compared to traditional antioxidants. It has been postulated that PT “chelator” might coordinate ferrous iron and shut down Fenton reaction^35^. But this seems unlikely in our scenario: first, the *in vitro* anti-oxidant activity of plasmid DNA was observed even without an iron catalyst (due to difficult manipulation of low concentration H_2_O_2_); second, the PT consumption was observed at the same level of molar equivalent of H_2_O_2_ in the extracted plasmid experiments, indicating that PT is consumable in the anti-oxidant process. There is no evidence that PT would be inert toward hydroxyl radical, irrespective of whether PT coordinates the metal ion or not. Moreover, the coordination of PS to Fe(II) was relatively weak – compared to Fe(II)-EDTA (IC50 = 1.27 ± 0.05 μg/ml at 12.5 μM [Fe^2+^]), the absorbance of Fe(II)-ferrozine complex did not change in the presence of PS from 0.01 to 5.00 mM (Fig. S6). It is likely that even a strong chelator like EDTA (ethylene di-amine tetra-acetic acid) cannot inhibit oxidative stress mimicking with Fenton chemistry. Probably this is due the high sensitivity of the biological system to trace amount of oxidative damage on the DNA molecule, and so PT-metal coordination is likely to be unimportant in its anti-oxidant activity. It should also be mentioned that both GSH and CYS exhibited severe side-effect at concentrations above 250.0 μM, that is, more ROS was generated (pro-oxidant effect). Indeed, hydroxyl radical - the dominant ROS product of Fenton reaction - is the most lethal to DNA molecule, responsible for the formation of G^•+^ radical cation and then resulting 8-OHdG^36^,^37^.

Regarding the issue of PT-DNA backbone cleavage during oxidation, we repeated LCMS analysis of GsA oxidation of our previous work^15^, using Fenton’s reagent, instead. Oxidant ABTS•+ resulted in the same product as the Fenton’s reagent, but no G_H_A was detected in the presence of excessive amount of oxidants. The G_H_A formation observed in the previous work was due to peroxyacetic acid (PAA) usage or high concentration of H_2_O_2_^15^,^16^. Importantly, the GsA oxidation led to only GA, no PSSP disulphide was observed as in the PS oxidation. In the absence of Fenton catalyst, no GA product was detected in the mixture of 200 μM H_2_O_2_ and 20 μM GsA after 30 min of incubation in the Tris-HCl buffer (pH = 7.4) (Fig. S7). Thus, the PT-DNA oxidation by hydroxyl radical likely resulted in a clean conversion to PO-DNA, i.e. the normal form of phosphate.

Therefore, the oxidation of phosphorothioate by ROS generated by Fenton’s reagent is different from free thiols. For example, CYS oxidation by H_2_O_2_ involves in a two-step reaction, forming sulfenic acid (CSOH) intermediate and then disulfide (CSSC), sulphenic, sulphinic or even sulphonic acid with increasing ROS concentrations^38^,^39^. The oxidation of phosphorothioate dinucleotide could desulfurize to normal phosphate^40^, in particular for the dilute PT encountering hydroxyl radical in a weak alkaline circumstance.

Owing to the experimental limitation, we distinguished the question whether the hydrogen-phosphonate species (PH) is an intermediate in the PS-PO conversion or a branched by-product of H_2_O_2_-dependent oxidation by quantum-chemistry calculations. As shown in Fig. 5, the PH generation needs two molecules of H_2_O_2_ with a higher reaction barrier, consistent with the HPLC-MS experimental observation in which the PH formation was highly dependent on the concentration of H_2_O_2_. The neural form of PH has two tautomeric structures, either as protonation on a phorsphorous center or as oxygen atom^41^. Hydrogen phosphonate would hydrolyze P-O bond into two parties at a rate of 3.2 × 10^5^ M/s at 25 °C^42^. Thus, unlike phosphate, hydrogen phosphonate hydrolyzes quickly and causes DNA backbone cleavage, as the *dnd*+ phenotype in PT-DNA gel electrophoresis.

Based on the quantum calculations, hydrogen-sulfur radical HS^•^ was generated in the PS-PO conversion (Fig. 5). Opposite to extremely oxidative hydroxyl radical, HS^•^ is extremely reductive! HS^•^ will degrade rapidly to sulphur (S_8_), sulfite (SO_3_^2−^) and sulfate (SO_4_^2−^), creating a specific reductive atmosphere in the surrounding compartment^43^,^44^.

At this point we tackle the question of how ROS invasion can precisely hit the PT site *in vivo* and *in vitro* when a PT site has an average scatter of 100~10000 nt. We believe that radical G-hopping in DNA oxidative damage helps to explain why the guanine-neighbouring PT site captures DNA damage efficiently. As shown in Fig. 6, both the HOMO and LUMO of GA anion and radical coincided each other in population, indicating that electron donation occurred on the guanine moiety and phosphate group in the oxidation of normal DNA, which is in agreement with the previous studies^15^. By contrast, the HOMO of GsA anion was consistent with the HOMO of GA and the SOMO of GsA radical in population, indicating that the guanine moiety of GsA is able to donate electron as well; however, the LUMO of GsA radical was computed to be dominant for the sulfur atom in population, indicating the likely formation of a phosphorothioate radical, which, as a result, keeps the guanine moiety intact in the PT-DNA oxidation. Such an electron transfer was further confirmed by the distributions of Mulliken charge and spin density of the two forms of GsA and GA, where the GA radical presented a significant charge separation between the nucleobases (+0.41 and +0.29) and phosphate (−0.97) groups with an unpaired electron on the guanine moiety (0.922), while the GsA radial only loses the negative charge (−1.296 → −0.253) and casts most of spin density (1.021) on the phosphorothioate group. We conclude that the phosphorothioate radical is hydrolyzed as via the same TS3 and TS4 transition states as shown in Fig. 5, leading to the formation of normal phosphate linkage. This also explains why the GsA was more reactive in scavenging ROS than PS, since the guanine moiety played a role in bridging electron transfer, and the relatively higher pH environment benefitted the formation of the [PS-OH]^•−^ pre-reaction state, which can also be accelerated in the presence of hydroxyl radical as an oxidant.

In summary, due to the exceptional chemical nature of phosphorothioate, *E. coli (S*^+^) possesses DNA-intramolecular PT antioxidant activity, which acted as a guard against oxidative stress via effectively scavenging hydroxyl radical (the most lethal species to genetic materials), decreased the intracellular ROS level, and therefore limited the 8-OHdG production and promoted the GSH:GSSG ratio, and thereby provided additional protection from DNA oxidative damage. The reductivity of phosphorothioate is powerful in protecting the most vulnerable moiety (guanine) of the DNA molecules, but not too intensive to induce a pro-oxidant side effect in cell.

**Figure 7:**
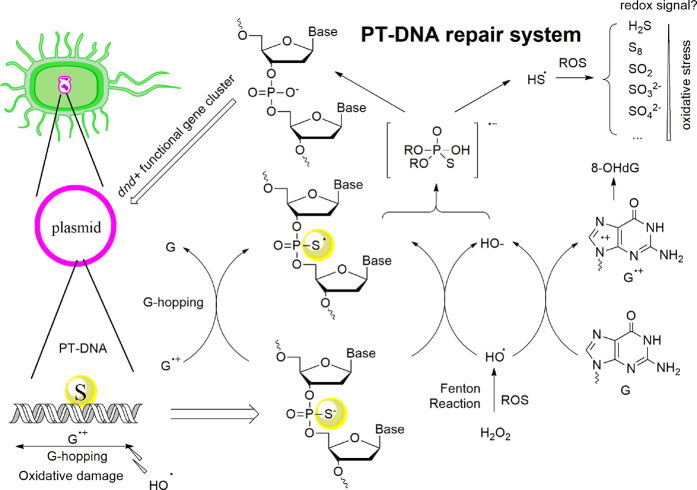
The proposed PT-DNA anti-oxidant mechanism, by which one equivalent of PS provides capacity larger than free thiol.

*In vitro* hydrogen phosphonate species was a branched by-product at the high H_2_O_2_ concentration. The PS-PO *in vivo* recycling does not require a hydrogen phosphonate intermediate, averting the dilemma of the PH-related DNA cleavage and genomic integrity discussed previously in the literatures. Figure 7 shows that the single-molecular PS-PO shortcut path led to the formation of highly reductive species, HS•, which as available for scavenging more surrounding ROS including O_2_^−•^ and H_2_O_2_. The oxidation of HS• towards sulphur (S_8_), sulfite (SO_3_^2−^) and sulfate (SO_4_^2−^) provided additional antioxidant capacity (see Fig. S9). Moreover, the computations clarified the reasons of why phosphorothioation frequently occurs at a guanine-rich location. Thus, at this stage, the biologically functional loop of anti-oxidative PT-DNA was closed with the *dnd*-gene-induced phosphate S-incorporation and the oxidative PS-PO recycling. It is also reasonable that eukaryote develops complicated *karyotheca* substructure tightly packing genetic materials away from mitochondria, while a prokaryote needs additional protector from oxidative metabolites. However, whether the PS-PO conversion cascades an *in vivo* ROS-signal to regulate PT-DNA expression in the ROS response needs further investigation. The novel mechanism of anti-ROS property of PT-DNA may be applicable in anti-tumor and anti-aging pharmaceutics.

## Methods

### General

*E. coli* DH10B harboring pJTU1238 was prepared as the *E. coli* (S^+^) strain, and the one with *dnd*C-disrupted pJTU1238 was used as the control in this work (namely the *E. coli* (S^−^) strain). The *E. coli* (S^+^) strain displayed the *dnd* phenotype in electrophoresis but the *E. coli* (S^−^) strain did not. All chemicals were of analytical grade and used without further purification. 8-OHdG ELISA kit was obtained from the Shanghai Enzyme-linked Biotechnology company. Plasmid Mini preparation kit was purchased from DingGuo Biotech company.

### DCFH-DA determination

In the *in vivo* ROS-level test, the 0.2 mg/ml DCFH-DA stock solution was added into the *E. coli* cultures to make the final concentration of 10 μM. After 30 min incubation at 37 °C, the suspension was washed with Tris-HCl (pH 7.4) and diluted to 3 × 10^8^ cfu. Then 150 μl samples in 96-well plate were treated with Fenton’s reagent. The intracellular ROS level was measured with the DCF fluorescence and the reaction condition was selected according to Fig. S1.

### 8-OHdG levels

The sample for the measurement of 8-OHdG levels was prepared in a four-hour incubation with Fenton’s reagent, sonication in ice-cold Tris-HCl, and centrifugation at 8000 G to remove cellular debris. In the 8-OHdG test, the supernatant was mixed with the stop reagent of the ELISA kit, and the absorbance at 450 nm was measured to compare to a calibration curve constructed with the standard 8-OHdG solutions at 2.5 to 40 pg/ml.

### Pre-oxidation of plasmid PT-DNA

This procedure was developed based on the ABTS+ oxidation of GsA, in which GA was determined as the only oxidative product by HPLC and LCMS. Accordingly, the plasmid PT-DNA extracted from the *dnd*+ (*S*^+^) *E. coli* was treated with 46.7 mM ABTS^•+^ and then purified as the pre-oxidized Ox(S+) plasmid DNA sample (Figs S2 and S7).

### Raman spectra

Raman spectra were recorded on a Bayspec Raman spectroscopy. For each kinetic measurement, H_2_O_2_ was added into the *S*^+^ or *S*^−^ *E. coli* strains washed by 10 mM PBS (*E. coli* OD_600_ ~0.75, peroxide ~30 mM, in 10 mM PBS). Then the time-resolved spectra were collected in a rate of one spectrum per 7 seconds. The Raman intensity at the wavenumber of 876 cm^−1^ (O-O vibration) was used to monitor the peroxide concentration^45^.

### CD spectra

CD spectra were measured on a Jasco J-815 spectropolarimeter. For each kinetic measurement, after running a blank spectrum with buffer (10 mM HEPES, pH 7.4), H_2_O_2_ (final concentration was 1 μM) was added into the plasmid DNA (final concentration was 63 μM), and then 30 spectra were recorded in a rate of 1 spectrum per minute.

### Gel electrophoresis

A typical gel electrophoresis was set on 1.5% agarose gels with 0.5 × TBE running buffer (45 mM Tris, 4.5 mM boric acid, 1 mM EDTA) at 100 V. Thereafter, the gels were stained with Geneview, visualized and digitally photographed by Alphalimager HP Gel Imaging system.

### Antioxidant assessment

Oxidant ABTS^•+^ was prepared by reacting ABTS with potassium persulfate according to the literature^46^. Using 46.7 μM ABTS^•+^ solution, the scavenging rate (SR) was calculated in percentage of its feature absorbance at 734 nm after 30-min reacting with different amounts of the antioxidant. IC_50_ value was estimated as the antioxidant concentration resulting in SR = 50%.

A similar method was used for the 0.1 mM DPPH oxidant. Instead, the feature absorbance at 520 nm was used to calculate the DPPH concentration^47^. IC_50_ value was estimated as the antioxidant concentration resulting in SR = 50%.

The *in vitro* ROS level was measured by the modified TBARS assay^48^. The mixture of antioxidant and Fenton’s reagent were vortexed and then settled for 30 min at 37 °C. TBA was dissolved in 10% trichloroacetic acid, with the final concentration of 0.67%. The solution was then heated in boiled water for 20 min. Finally the amount of TBARS formed in each sample was assessed with the absorbance at 532 nm.

The ferric-ferrous conversion was monitored with a modified colorimetric method, based on the absorbance of the Fe(II)-Ferrozine complex at wavelength of 562 nm.

### Electrochemistry

Electrochemical measurement was performed with a CHI 660D electrochemical analyser, with a three-electrode system, *i.e.* a saturated AgCl electrode, a platinum wire, and a platinum disc electrode used as the reference, auxiliary, and working electrodes, respectively.

### HPLC-MS analysis

The phosphorothioate oxidation was carried out in a mixed solution of 25 μM PS or 20 μM GsA-Rp and GsA-Sp with different concentrations of oxidants (H_2_O_2_ or Fenton reagents), respectively. The mixtures were settled for 2 hours in the dark at room temperature before the HPLC-MS analysis. In the analysis, 10 μl of reaction sample was loaded on Agilent’s C18 reversed phase column (150 × 4.6 mm, 1.5 μm), water and acetonitrile with 0.1% acetic acid were used as eluent A and eluent B in HPLC, respectively, and the flow rate was 0.3 ml/min with a gradient elution B from 5 to 95% in 10 min. Drying gas flow: 8 L/min; nebulizer pressure: 35 psi; drying gas temperature: 320 °C; capillary voltage: 3500 V (using the positive detection mode and scanning between 50 and 900 m/z for mass spectrometer analysis).

### Quantum chemical calculation

All DFT (density functional theory) calculations were carried out with Gaussian 09 package^49^. Geometries were first optimized at the B3LYP/6-31 + G* level. In a typical calculation, the M06 and B3LYP levels of theory with the larger basis sets of 6-311 + G(d, p) were used to evaluate the geometric and energetic results in the gas phase. Solvent effect was considered with single point calculations of a continuum model (SMD) on reactants, transition states, intermediates, and products.

## Additional Information

**How to cite this article**: Wu, T. *et al*. Mechanistic Investigation on ROS Resistance of Phosphorothioated DNA. *Sci. Rep.* **7**, 42823; doi: 10.1038/srep42823 (2017).

**Publisher's note:** Springer Nature remains neutral with regard to jurisdictional claims in published maps and institutional affiliations.

## Supplementary Material

Supplementary Information
