# Supplementary material for: Mechanistic Investigation on ROS Resistance of Phosphorothioated DNA
Source: Sci Rep. 2017 Feb 20;7:42823. doi: 10.1038/srep42823 (PMC5316992; doi:10.1038/srep42823)
Supplement: Supplementary Information [file srep42823-s1.doc]

**Supplementary Information**

**Mechanistic Investigation on ROS Resistance of Phosphorothioated DNA**

Tingting Wu,^1^ Qiang Huang,^1^ Xiao-Lei Wang,^1^ Ting Shi,^1^ Linquan Bai,^1^ Jingdan Liang,^1^ Zhijun Wang,^1^ Zixin Deng,^1^ Yi-Lei Zhao^1^, *

*^1^State Key Laboratory of Microbial Metabolism, Joint International Research Laboratory of Metabolic & Developmental Sciences, School of Life Sciences and Biotechnology, Shanghai Jiao Tong University, Shanghai, 200240, China*

*To whom correspondence should be addressed:

**Prof. Yi-Lei Zhao**

School of Life Sciences and Biotechnology

Shanghai Jiao Tong University

800 Dongchuan Road, Shanghai 200240, China

Tel/Fax: +86-21-34207190;

Email: [yileizhao@sjtu.edu.cn](mailto:yileizhao@sjtu.edu.cn)

**Contents**

**Figure S1.** The intracellular ROS level against Fenton’s regents…………………...S3

**Figure S2.** The image of gel electrophoresis in iodine assay….…………………….S4

**Figure S3:** The Fe(II)-ferrozine assay for testing reaction kinetics…...…………….S5

**Figure S4:** The Fe(II)-ferrozine assay for testing ferrous yields…………………….S6

**Figure S5**. The condition tests for Fenton’s digestion………………………………S7

**Figure S6**. The UV-vis spectra for testing metal-ion interaction……………………S8

**Figure S7:** The HPLC-MS characterization for GsA oxidation……………………..S9

**Figure S8**. Calculated structures for the PS-H_2_O_2_ oxidation……………………….S10

**Figure S9**. The ion chromatography for the PS-H_2_O_2_ oxidation……..…………….S11

**Table S1.** The intracellular ROS level over the oxidation time…………….……...S12

**Table S2.** The intracellular 8-OH-dG level against H_2_O_2_ concentrations………….S13

**Table S3.** The intracellular GSH level against H_2_O_2_ concentrations………...…….S14

**Table S4.** The intracellular GSSG level against H_2_O_2_ concentrations………...…..S15

**Table S5.** The nicked DNA quantitation in gel electrophoresis……………...........S16

**Table S6.** DPPH and ABTS^•+^ IC_50_ of PS, vitC, CYS, and GSH…………………...S17

**Table S7.** Cartesian coordinates of TSs 1-4 …………………..…………………...S18

**
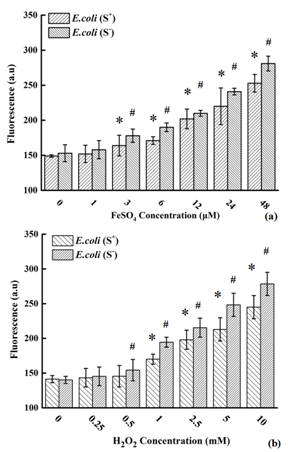
**

**Figure S1.** The DCF fluorescent intensity (representing intracellular ROS level) of the *E. coli* (S^+^) and (S^-^) strains, treated with the different concentrations of H_2_O_2_ (0, 0.25, 0.5, 1, 2.5, 5, and 10 mM) and FeSO_4_ (0, 1, 3, 6, 12, 24, and 48 μM). (Material and methods 2.2. excitation: 485 nm, emission: 528 nm)


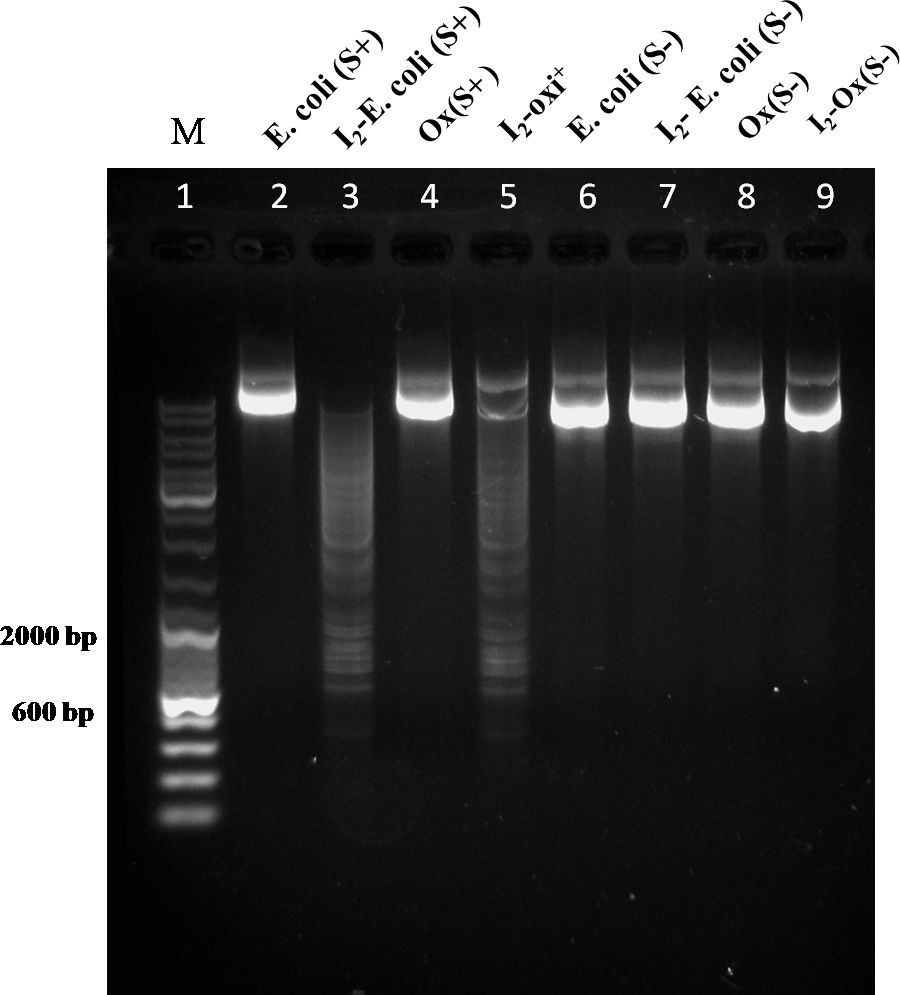


**Figure S2.** The image of gel electrophoresis of plasmid DNAs by the iodine assay. Lane 1, Marker;

Lane 2, PT-DNA from *E. coli* (S^+^), 63 ng/μl;

Lane 3, PT-DNA treated with 0.5 mM I_2_;

Lane 4, Ox(S^+^), pre-oxidized PT-DNA, 63 ng/μl;

Lane 5, Ox(S^+^) treated with 0.5 mM I_2_;

Lane 6, control DNA from *E. coli* (S^-^), 63 ng/μl;

Lane 7, control DNA treated with 0.5 mM I_2_;

Lane 8, Ox(S^-^), “pre-oxidized” DNA;

Lane 9, Ox(S^-^) treated with 0.5 mM I_2_.

All the samples were incubated for 30 min at room temperature with or without I_2_ before gel electrophoresis.


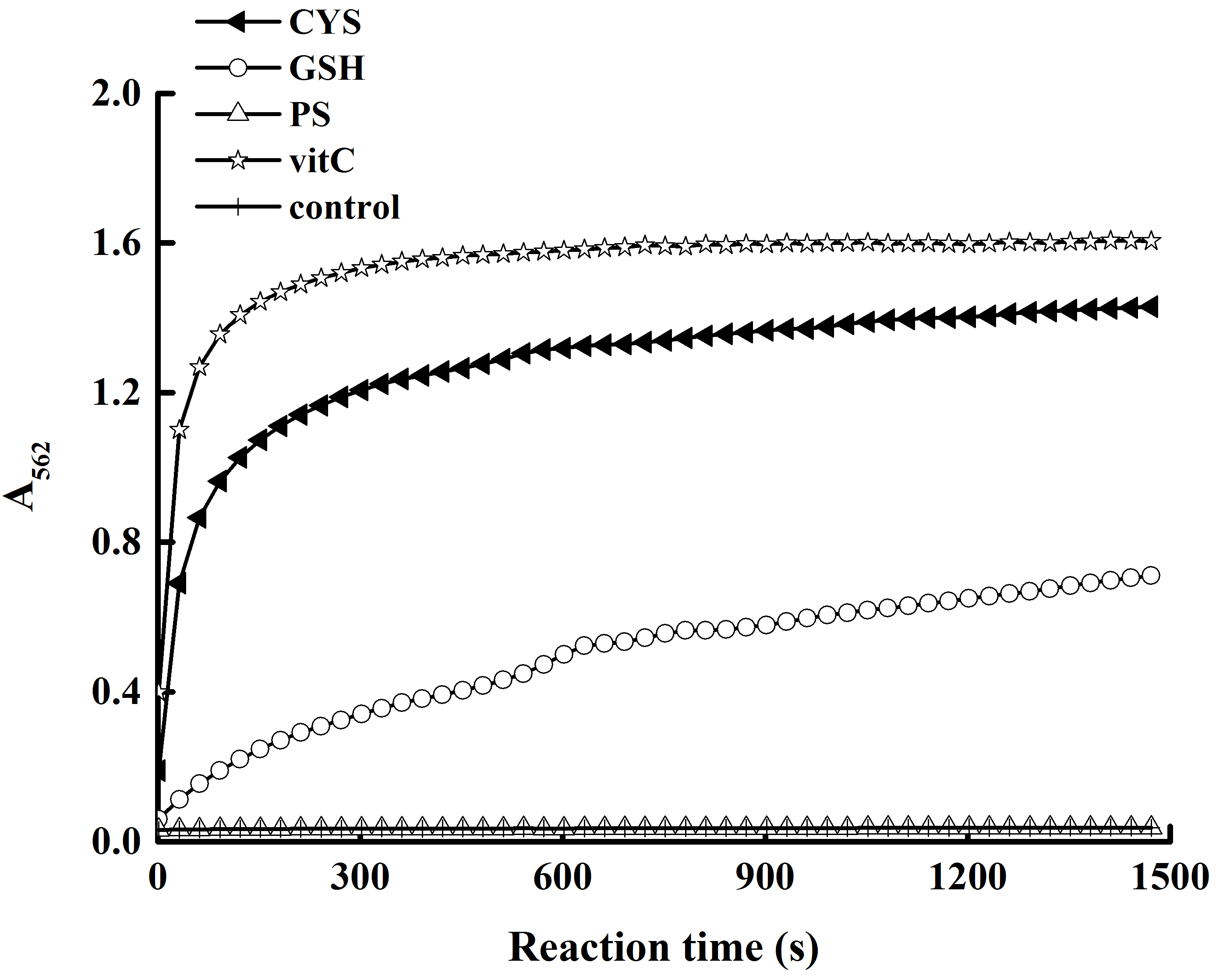


**Figure S3:** The time-resolved absorbance at wavelength of 562 nm in Fe(II)-ferrozine assay. Reaction mixture: 50 μM antioxidant, 50 μM FeCl_3_, and 1.0 mM ferrozine (as chromogenic agent of Fe(II) ion).


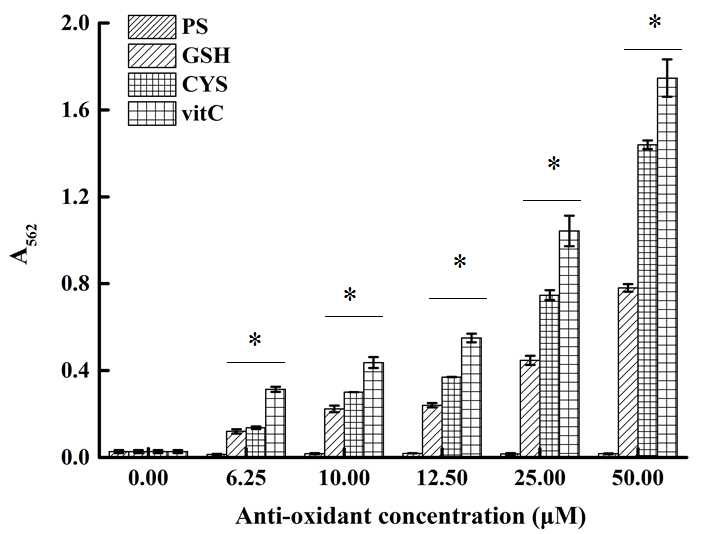


**Figure S4:** Fe(II) production in Fe(II)-ferrozine assay, with antioxidant concentrations of 0.00, 6.25, 10.00, 12.50, 25.00, and 50.00 μM antioxidants of PS, GSH, CYS, and vitC.

**
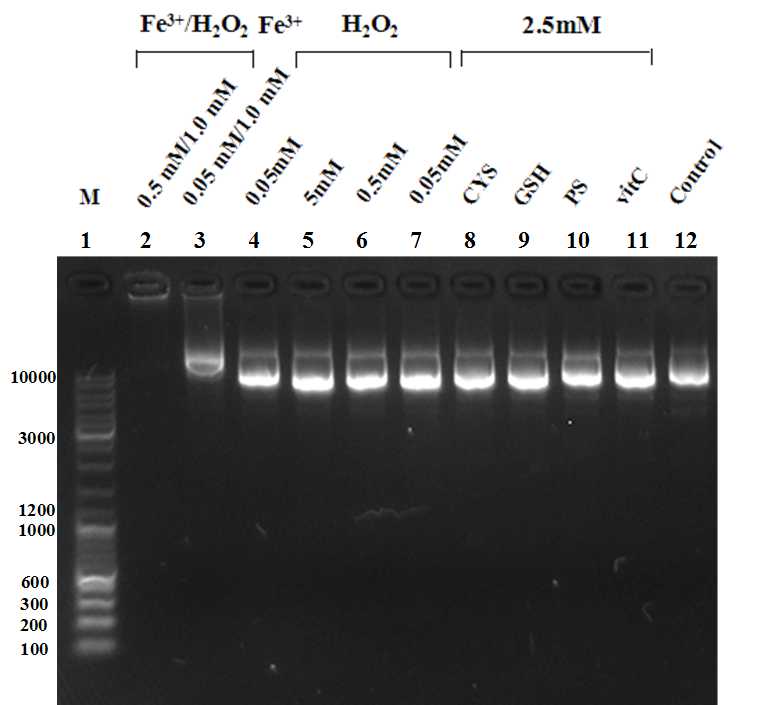
**

**Figure S5**. The image of gel electrophoresis of *E. coli* (S^-^) plasmid DNA (50 ng) upon the following treatments (20 min).

Lane 1, DNA marker;

Lane 2, 0.5 mM FeCl_3_ plus 1.0 mM H_2_O_2_ in 2.0 mM HCl;

Lane 3, 0.05 mM FeCl_3_ plus 1.0 mM H_2_O_2_ in 2.0 mM HCl;

Lane 4, 0.05 mM FeCl_3_;

Lane 5, 5mM H_2_O_2_;

Lane 6, 0.5 mM H_2_O_2_;

Lane 7, 0.05 mM H_2_O_2_;

Lane 8, 2.5 mM CYS;

Lane 9, 2.5 mM GSH;

Lane 10, 2.5 mM PS;

Lane 11, 2.5 mM vitC;

Lane 12, *E. coli* (S^-^) plasmid DNA (control).

**Figure S6**. The UV-vis spectra of 150 μM FeSO_4_ (black), 150 μM PS (red), 150 μM EDTA (purple), FeSO_4_ plus PS (blue), and FeSO_4_ plus EDTA (green).

The Fe(II)-EDTA complexation showed typical spectral shift caused by metal-ion interaction, while no significant change was detected in the mixture of FeSO_4_ plus PS.

**
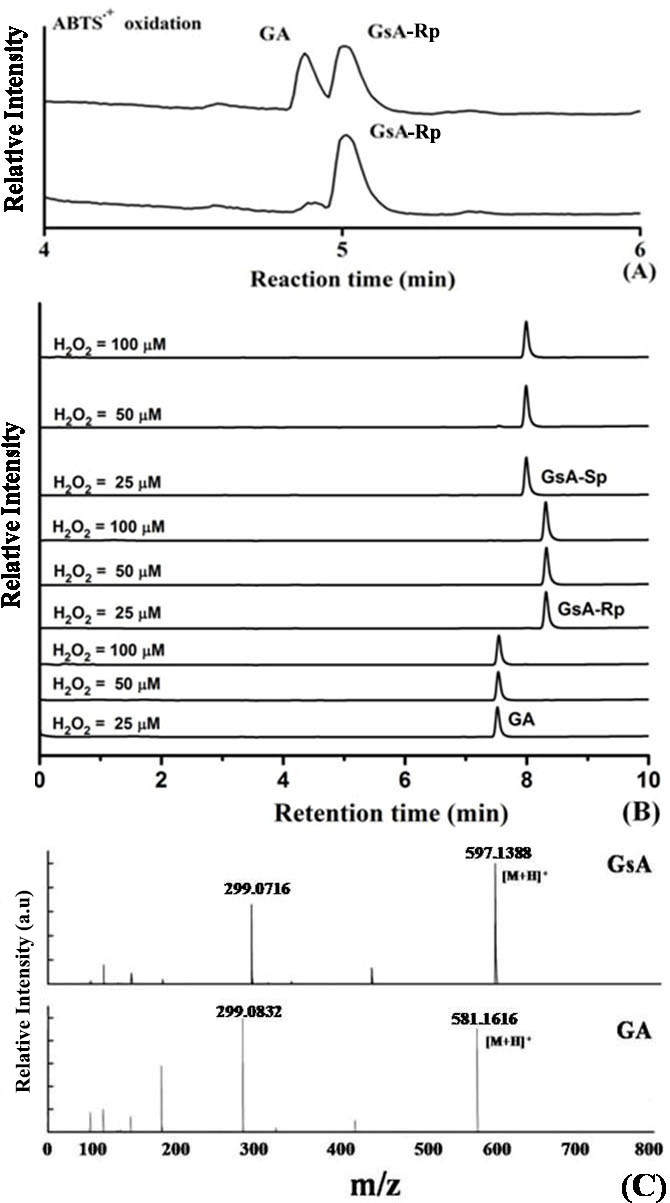
**

**Figure S7:** The HPLC-MS characterization for GsA oxidation: (A) the HPLC results of 20 µM GsA with 40 µM ABTS^.+^ in pH=6.5 buffers. (B) the HPLC results of 20 μM GsA-Rp, GsA-Sp, GA with different concentrations of H_2_O_2_ (25 µM, 50 µM, 100 µM). (C) the high-resolution mass spectra of GsA. (Material and methods 2.9.)

Experimental details: 20 µl of reaction sample was loaded on C18 reversed phase column (150 × 4.6 mm, 1.5 µm); water and acetonitrile with 0.1 % acetic acid were used as eluent A and eluent B; a gradient elution B from 5 to 95% in 9 min; the flow rate of 1.0 ml/min.


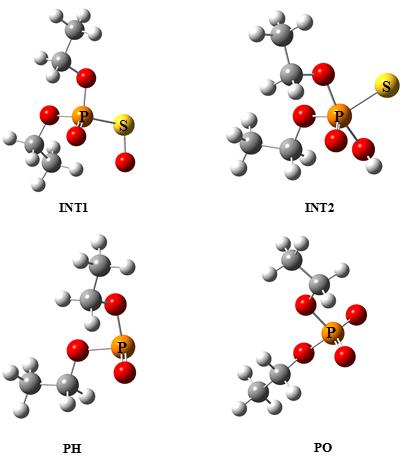


**Figure S8**. Structure of products (PH and PO) and intermediate (INT) in the oxidation of PS. Geometries were optimized at the B3LYP/6-311++G(d,p) level of theory in the gas phase.


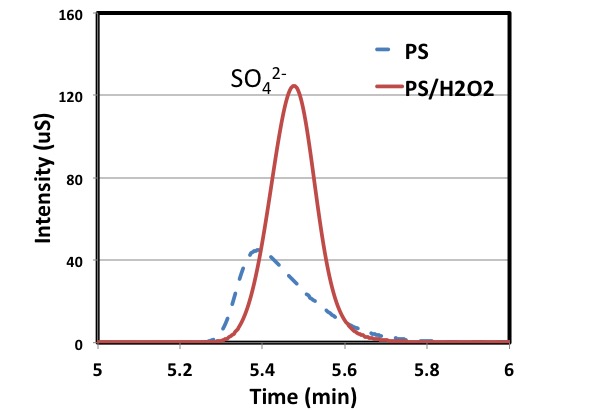


**Figure S9**. The ion chromatography of 50 mM diethyl phosphorothioate (PS) before and after the oxidation by Fenton agent. (dilution factor: 10, and injection volume: 25.00 uL.) The oxidation of 50 mM PS produced a considerable amount of sulfate in the presence of 1000 mM H_2_O_2_ and 5 mM FeCl_2_, increasing from 4.2 to 7.8 mM in 48 hour.

**Table. S1.** The DCF fluorescent intensity (corresponding to the intracellular ROS level) against the treatment time, with 0.5 mM H_2_O_2_ plus 6.0 µM FeSO_4_.

|  | **Fluorescent intensity** | |
| --- | --- | --- |
| Time (min) | *E. coli (S^+^)* | *E. coli (S^-^)* |
| 0 | 175 ± 7 | 169 ± 5 |
| 1 | 185 ± 7 | 198 ± 13 |
| 5 | 218 ± 8 | 237 ± 15 |
| 10 | 204 ± 20 | 240 ± 7 |
| 15 | 182 ± 13 | 245 ± 14 |
| 20 | 225 ± 14 | 277 ± 17 |
| 25 | 248 ± 14 | 337 ± 17 |

**Table S2.** The UV-vis absorbance (corresponding the intracellular 8-OH-dG level) against H_2_O_2_ concentration, treated for 4 hours with the coexistence of 6.0 µM FeSO_4_.

|  | **Absorbance of 8-OH-dG** | |
| --- | --- | --- |
| H_2_O_2_ (mM) | *E. coli (S^+^)* | *E. coli (S^-^)* |
| 0.0 | 0.16 ± 0.01 | 0.13 ± 0.03 |
| 0.5 | 0.18 ± 0.03 | 0.18 ± 0.01 |
| 1.0 | 0.18 ± 0.06 | 0.23 ± 0.02 |
| 1.5 | 0.23 ± 0.04 | 0.27 ± 0.03 |
| 2.0 | 0.25 ± 0.01 | 0.37 ± 0.02 |

**Table S3.** The UV-vis absorbance (corresponding the GSH level) against H_2_O_2_ concentration, treated for 4 hours with the coexistence of 6.0 µM FeSO_4_.

|  | **Absorbance (a.u) of GSH** | |
| --- | --- | --- |
| H_2_O_2_ (mM) | *E. coli (S^+^)* | *E. coli (S^-^)* |
| 0.0 | 0.60 ± 0.03 | 0.77 ± 0.02 |
| 0.5 | 0.72 ± 0.02 | 0.70 ± 0.04 |
| 1.0 | 0.77 ± 0.01 | 0.65 ± 0.01 |
| 1.5 | 0.82 ± 0.01 | 0.60 ± 0.05 |
| 2.0 | 0.85 ± 0.02 | 0.58 ± 0.02 |

**Table S4.** The UV-vis absorbance (corresponding the GSSG level) against H_2_O_2_ concentration, treated for 4 hours with the coexistence of 6.0 µM FeSO_4_.

|  | **Absorbance (a.u) of GSSG** | |
| --- | --- | --- |
| H_2_O_2_ (mM) | *E. coli (S^+^)* | *E. coli (S^-^)* |
| 0.0 | 0.45 ± 0.04 | 0.42 ± 0.03 |
| 0.5 | 0.44 ± 0.05 | 0.46 ± 0.02 |
| 1.0 | 0.43 ± 0.04 | 0.47 ± 0.02 |
| 1.5 | 0.42 ± 0.03 | 0.51 ± 0.04 |
| 2.0 | 0.40 ± 0.05 | 0.53 ± 0.02 |

**Table S5.** The *in vitro* nicked DNA in plasmid DNA gel electrophoresis with different concentrations of H_2_O_2_.

|  | **Nicked DNA (%)** | | |
| --- | --- | --- | --- |
| H_2_O_2_ (μM) | *E. coli (S^+^)* | *Ox(S^+^)* | *E. coli (S^-^)* |
| 0.0 | 11.4 ± 1.4 | 13.5 ± 1.6 | 10.9 ± 1.7 |
| 0.5 | 13.0 ± 1.6 | 13.0 ± 2.0 | 14.9 ± 2.9 |
| 1.0 | 14.1 ± 1.8 | 23.0 ± 1.0 | 19.8 ± 1.1 |
| 1.5 | 24.2 ± 1.0 | 38.0 ± 1.4 | 28.6 ± 1.7 |

**Table S6.** DPPH and ABTS^•+^ IC_50_ of PS, vitC, CYS, and GSH.

| **Compounds** | **IC_50_(µM)^a^** | |
| --- | --- | --- |
|  | **DPPH^•^** | **ABTS^•+^** |
| **PS** | 230.7 ± 8.1 | 42.3 ± 1.2 |
| **vitC** | 25.3 ± 0.4 | 7.1 ± 0.1 |
| **CYS** | 31.2 ± 0.2 | 5.9 ± 0.1 |
| **GSH** | 36.5 ± 1.5 | 5.1 ± 0.2 |

^a^: IC_50_ value was the concentration of the tested compounds at 50 % scavenging rate of DPPH**^•^** or ABTS**^•+^**

**Table S7.** Cartesian coordinates of the transition states optimized at the B3LYP/6-311++G(d,p) level of theory

TS1

| S | -0.51110500 | -1.88242600 | 0.61657400 |
| --- | --- | --- | --- |
| O | -2.07602500 | -1.04540800 | -0.61832500 |
| H | -1.53066700 | -0.98347500 | -1.41166500 |
| O | -3.35636000 | -0.26941500 | -1.85017700 |
| H | -4.02692600 | -0.88459800 | -1.54267200 |
| P | 0.67987200 | -0.24144900 | 0.74381000 |
| O | -0.16147700 | 1.04449100 | 0.23775400 |
| O | 1.70756400 | -0.34154500 | -0.51453000 |
| C | -1.28079000 | 1.49615600 | 1.00017900 |
| C | -2.23121300 | 2.20279800 | 0.07204900 |
| H | -0.90876100 | 2.15025600 | 1.80252400 |
| H | -1.77771800 | 0.63388400 | 1.46420900 |
| H | -3.06154400 | 2.63247700 | 0.64523800 |
| H | -1.71963200 | 3.02002300 | -0.44951300 |
| H | -2.63959200 | 1.49393200 | -0.66003900 |
| C | 2.71202300 | 0.65232700 | -0.64336900 |
| C | 3.58428200 | 0.27443900 | -1.81184300 |
| H | 3.29287200 | 0.71090600 | 0.28754600 |
| H | 2.23993600 | 1.63232700 | -0.80390000 |
| H | 4.37940500 | 1.01375000 | -1.95677800 |
| H | 4.04395900 | -0.70425000 | -1.64338100 |
| H | 2.99103600 | 0.21667300 | -2.72934700 |
| O | 1.38522600 | -0.00633300 | 2.02944700 |

**TS2**

| S | -0.74681000 | 0.34624700 | -1.69842200 |
| --- | --- | --- | --- |
| O | -2.29463300 | 0.43965500 | -1.60336400 |
| H | 0.00130100 | 1.70214600 | 0.33746800 |
| O | 0.51412300 | 2.05553300 | -0.40417800 |
| P | -0.19357000 | -1.08881700 | -0.20026800 |
| O | -0.40830900 | -0.36719200 | 1.25516200 |
| O | 1.40738200 | -1.20494400 | -0.25712500 |
| C | -1.66079400 | -0.49262700 | 1.93382900 |
| C | -2.57423200 | 0.65803800 | 1.59204800 |
| H | -1.41717000 | -0.51317700 | 3.00266800 |
| H | -2.11572700 | -1.45549300 | 1.66978400 |
| H | -3.52792700 | 0.55370400 | 2.12267400 |
| H | -2.11988600 | 1.61028900 | 1.89034300 |
| H | -2.76816400 | 0.68415100 | 0.50946500 |
| C | 2.34755200 | -0.25727800 | 0.26984300 |
| C | 3.69990300 | -0.91637300 | 0.24426600 |
| H | 2.05033600 | 0.01587300 | 1.28911600 |
| H | 2.31797500 | 0.65957700 | -0.32576300 |
| H | 4.45900700 | -0.22890000 | 0.63145900 |
| H | 3.70515100 | -1.82498600 | 0.85489400 |
| H | 3.97351400 | -1.19312100 | -0.77874600 |
| O | -0.86537000 | -2.40298600 | -0.26867100 |
| O | 1.42473400 | 3.09746200 | 0.60590300 |
| H | 1.19611300 | 3.89146500 | 0.11368400 |

TS3

| P | -0.16222200 | -0.50580500 | 0.27541400 |
| --- | --- | --- | --- |
| O | 1.43990200 | -0.41780500 | -0.21300100 |
| O | -0.57585300 | 0.86632400 | -0.51880600 |
| C | 2.25357400 | 0.57122300 | 0.35984800 |
| H | 1.81356800 | 1.57032900 | 0.19733100 |
| H | 2.32032200 | 0.42417400 | 1.44856000 |
| C | -1.62975400 | 1.68799700 | -0.06125500 |
| H | -2.57728600 | 1.34747000 | -0.49484500 |
| H | -1.72405300 | 1.60243400 | 1.02928700 |
| O | -2.26774200 | -0.93539900 | 0.25686300 |
| O | -0.14535900 | -0.37058700 | 1.76375300 |
| S | -0.53198300 | -2.24981700 | -0.74621500 |
| C | 3.61719800 | 0.48951600 | -0.28318100 |
| H | 4.05516100 | -0.50104200 | -0.12166600 |
| H | 3.54016300 | 0.64989900 | -1.36353200 |
| H | 4.29547800 | 1.24278600 | 0.13490500 |
| C | -1.30578200 | 3.10616000 | -0.46602800 |
| H | -2.10164700 | 3.79141700 | -0.15133800 |
| H | -0.36531300 | 3.43107200 | -0.00690600 |
| H | -1.19360700 | 3.18189000 | -1.55291300 |
| H | -2.33673700 | -1.42591800 | 1.08256200 |

**TS4**

| P | 0.24321300 | -0.54657300 | 0.13314800 |
| --- | --- | --- | --- |
| O | 0.83985000 | 0.95892700 | -0.33513800 |
| O | -1.20746500 | -0.11900900 | -0.63036300 |
| C | 0.06903000 | 2.11497800 | -0.07560400 |
| H | -0.70729700 | 2.23662700 | -0.84398300 |
| H | -0.44369000 | 2.02055600 | 0.89372300 |
| C | -2.38860800 | -0.78838500 | -0.28606800 |
| H | -2.76842800 | -1.33343300 | -1.16563300 |
| H | -2.20867000 | -1.53086600 | 0.50209500 |
| O | 0.05814000 | -2.06997000 | -0.60170300 |
| O | -0.04357600 | -0.60454600 | 1.59959700 |
| S | 2.50028100 | -1.07702600 | -0.04832800 |
| H | 0.72911300 | -2.64619800 | -0.21545200 |
| C | 1.01260900 | 3.29434400 | -0.06715300 |
| H | 1.76599500 | 3.17814700 | 0.71814800 |
| H | 1.53622200 | 3.37111700 | -1.02590200 |
| H | 0.46548400 | 4.22891700 | 0.10613500 |
| C | -3.40061900 | 0.22945200 | 0.19443500 |
| H | -4.36910800 | -0.23763800 | 0.41359700 |
| H | -3.02931500 | 0.71238000 | 1.10519600 |
| H | -3.55307100 | 1.00586600 | -0.56470100 |
